# Online Presence of the Funeral Industry: The Example of the Quebec Federation of Funeral Cooperatives

**DOI:** 10.1177/00302228221111936

**Published:** 2022-06-29

**Authors:** Elisabeth Beaunoyer, Alexandre Guitton, Matthieu J. Guitton

**Affiliations:** 1 Faculty of Medicine, 4440 Université Laval, Quebec City, QC, Canada; 2 Faculty of Nursing, 4440 Université Laval, Quebec City, QC, Canada; 3 CERVO Brain Research Center, Quebec City, QC, Canada; 4 Université Clermont-Auvergne, CNRS, Mines de Saint-Etienne, Clermont-Auvergne-INP, LIMOS, 63000 Clermont-Ferrand, France

**Keywords:** community, cyberthanatology, funeral industry, social media, thanatology

## Abstract

The digitalization of modern societies has offered new tools for funeral industries to reach the communities they serve, ranging from using technologies in funeral planning, in commemoration of the dead, or to support the bereaved. The COVID-19 pandemic only pushed this need for online presence of the funeral industries further. We explore the digitalization of funeral industries through the example of Quebec (Canada), where many funeral institutions are regrouped under a federation of funeral cooperatives. We analyze how this influences the delivery of funeral services and allows the development of common services answering various needs of the population (e.g., a funding program for youth funerals, an ecological memorialization program, online grief support). Finally, we discuss how the federation’s online presence contributes to its mission, and more largely whether it changes the perception of the industry.

## Introduction

Dealing with death has always been a challenging issue for humans. In an attempt to cope with the unavoidable, humans have developed funeral rituals, therefore transferring part of the grieving burden from a personal to a societal perspective responsibility (Metcalf & Huntington, 1991; Romanoff & Terenzio, 1998). In modern Western societies, an important part of the practical aspects of these rituals is devolved to funeral institutions (Beard & Burger, 2017). With Internet as a flagship, the ubiquity of communication technologies has impacted all aspects of life, and death is not an exception. Indeed, the interactions between the living and the dead have been increasingly reshaped by the overwhelming impact of emerging technologies, leading to changes of the global context in which death and bereavement experiences occur, and to the embedment of end-of-life experiences with digitalized spaces (Beaunoyer, Hiracheta Torres, et al., 2020; Beaunoyer & Guitton, 2021). Although thanatologists and other death professionals remain essential in a digitalized society, even if only for the disposal of the physical body, technology-based emerging media offer new means of conducting digital rituals as well as alternative possibilities that could challenge the role of the funeral services (Nansen et al., 2017). Indeed, the digital revolution further imposed pressures on the funeral industry to adapt themselves by using technologies in funeral planning, in commemoration of the dead and for supporting the bereaved (Beard & Burger, 2017; Beaunoyer & Guitton, 2021; Nansen et al., 2017). While this adaptation can stimulate the development of digital services such as cyberfunerals, online memorials, or augmented reality (Arnold et al., 2018), the digital funeral industry does not rely solely on how technologies are used to support funeral planning for bereaved and family, and further the commemoration of the dead. Instead, technologies can also be used to improve marketing strategies. Indeed, as most people refer to Internet to seek health information (Fox, 2011; Cyrus, 2014), the same phenomenon is observed when looking for practical information, products, and services (Bernier, 2017; Silver et al., 2019; Turner & Rainie, 2020). Therefore, another important feature to investigate is the online presence of the funeral industry, meaning the display of the establishment name online, through a website, social media, or blogs, in order to give opportunities for customers who prefer to compare prices and services digitally to learn about those before reaching out to the services providers (Beard & Burger, 2020) – in other words, how they make themselves shown and known.

Based on these observations, the Quebec federation of funeral cooperatives represents an extremely interesting case study. Indeed, this federation of funeral cooperative gathers several advantages. First, it covers a territory which, while not being strictly superimposed by the borders of the province of Quebec, can nonetheless be delineated relatively easy – notably by the language used (French vs. surrounding English). Second, this territory does not only have a geographical reality, but also a cultural one. Third, while there are independent funeral companies in Quebec, the federation nonetheless gathers an important group of participating funeral homes, with 21 cooperatives, thus providing some interesting generalizing potential. Fourth, the federation membership covers a majority of the concerned territory (participating cooperatives belonging to 13 of the 17 administrative regions of the province), including both urban and rural areas. Finally, as we will discuss below, the federation and its members have an important online presence. Although the cooperatives in the network only represent a portion of the funeral services companies operating in the province of Quebec, the federation nonetheless is an interesting case study model, given its popularity.

In this study, the online presence of the Quebec federation of funeral cooperative was assessed qualitatively using a virtual anthropology approach (Hine, 2000; Mann & Stewart, 2000; Guitton, 2011; 2012). All of the cooperatives’ websites (21) and the federation main website (1) were explored thoroughly, as well as related material (e.g., forums, Facebook pages). Furthermore, the presence of the Quebec federation of funeral cooperative in online media was also observed. The multiplication, variety, and diversity of online sources allowed the information to be triangulated, providing a rich and robust corpus for qualitative analyses (Marcus, 1995; Mann & Stewart, 2000). All of the three investigators involved were native in French and fluent in English. In order to minimize the occurrence of potential cultural biases, the investigators brought different cultural perspective, as one of the three investigators was a French Canadian native, a second never lived in Canada, and the third was an immigrant to French Canada. Based on these in-depth qualitative analyses, this paper will analyze the federation’s online presence and how it contributes to its mission of bringing back a sense of community, and therefore of life, to something that was traditionally perceived as morbid. More largely, we will reflect on how this online presence can contribute to changing the perception of a profit-driven funeral industry by promoting grief and death literacy locally.

## Online identity of a unique institution

### The context of French Canadian funeral industry

Funeral cooperatives are quite present in Canada, particularly in the province of Quebec (Tessier, 2007; Fédération des coopératives funéraires du Québec, n.d.-a). In the 1980s and the 1990s, the centralization of ownership of funeral institutions in North America resulted in a handful of transnational companies running most funeral homes (Arnold et al., 2018; Northcott & Wilson, 2017). However, the French-speaking portion of Canada, particularly the province of Quebec, appeared to have been partially refractory to this phenomenon (Tessier, 2007). Indeed, an exhaustive mapping of online resources related to death and grief revealed that a higher number of French funeral websites were found originating from Canada than any other countries in which the searches took places, including France (Beaunoyer, Hiracheta Torres, et al., 2020). This suggests that either the funeral industry of the French portion of Canada is less centralized than elsewhere in North America or Western Europe, or that there is some degree of cultural differences in the way funeral institutions present themselves online. More likely, this might well be due to a combination of these two factors.

In the province of Quebec, the funeral industry comprises more than 200 funeral services companies, divided in three models including independent family businesses, transnational private companies, and funeral cooperatives (Ministère de la santé et des services sociaux, 2022; Fédération des coopératives funéraires du Québec, 2013). Interestingly, 21 funeral cooperatives are regrouped under a federation of funeral cooperatives (“*Fédération des coopératives funéraires du Québec*”; FCFQ, https://www.fcfq.coop/) totalizing more than a hundred service points, mainly scattered throughout the south of the province. Although, this network represents approximately 10% of the funeral services companies in the province, they had a market share of 18% in 2017 (Fédération des coopératives funéraires du Québec, n.d.-a). Some of these cooperatives are located in the province’s largest cities including both Montreal and Quebec City, while others are located in rural or remote areas. Of note, the federation also includes one auxiliary member (“*Résidence funéraire de Lanaudière*”) and few associate cooperatives from other Canadian provinces (i.e., New Brunswick, Ontario, and Prince Edward Island), and even from other countries (i.e., the United States, Peru, Costa Rica, and France). The federation’s cooperatives follow the Statement on the Cooperative Identity, the values, and the principles from the International Cooperative Alliance (ICA). According to the ICA, “a cooperative is an autonomous association of persons united voluntarily to meet their common economic, social, and cultural needs and aspirations through a jointly-owned and democratically-controlled enterprise” (International Cooperative Alliance, n.d.). Core values of the cooperative movement are “self-help, self-responsibility, democracy, equality, equity, and solidarity” (International Cooperative Alliance, n.d.). These values are enacted through seven principles including democratic member control, education training and information, cooperation among cooperatives, and concern for the community. Consequently, this model presents multiple characteristics influencing the delivery of funeral services that allows the development of common services answering various needs of the communities they serve.

The results from a quantitative study that aimed to describe the grief and bereavement websites landscape revealed that a small proportion of websites retrieved from a Google search pertain to the funeral industry. Indeed, extensive searches in the three most widely spoken Indo-European languages (English, French, and Spanish) unveiled 136 funeral institution websites, representing 5.25% of all websites found (Beaunoyer, Hiracheta Torres, et al., 2020). Interestingly, over two thirds of these websites were in French, and 78 websites were originating from Canada (Beaunoyer, Hiracheta Torres, et al., 2020). Among the 78 French Canadian funeral institutions websites, 14 websites were part of the Quebec federation of funeral cooperatives. Further investigations of the federation revealed that seven additional cooperatives were not identified in the sample of this original study – yet, have been included in the analyses presented here.

### Online presence: Visual identity and aesthetics

**Figure 1.**
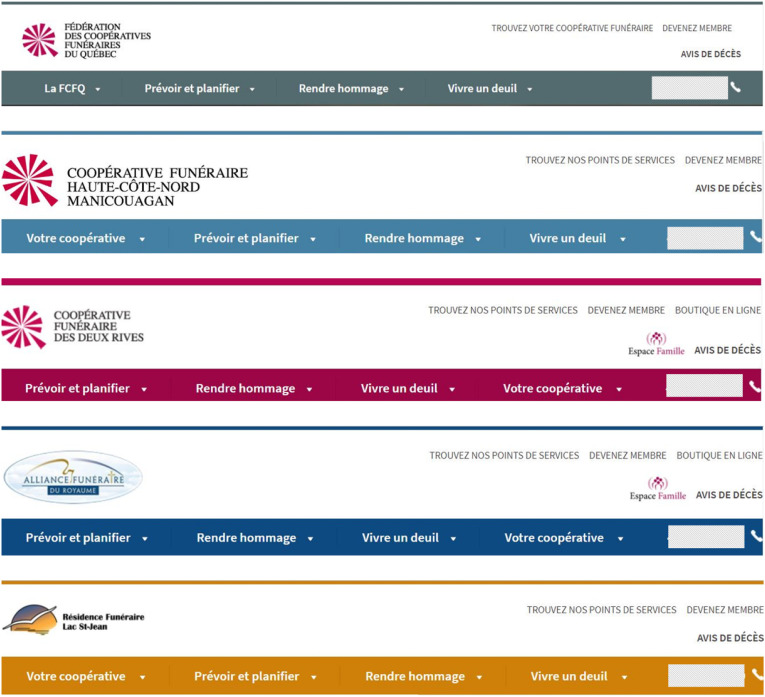
Examples of shared elements of web design. Shown is a photo-edition of homepage banners taken from the websites of the federation and of some of its members. A common framework is easily identifiable across the different examples, although the color code varies for each member of the federation. The top line displays the federation main website (https://fcfq.coop) with the federation’s logo on the left. The second and third lines display examples of individual members reproducing the same layout, including a similar logo than the federation. The two lower lines present examples of individual members reproducing the same layout as the federation, but with a fully different logo not bearing similitude with the federation’s logo. Interestingly, all examples (including those not having the same logo) presented the same section headings albeit their order often varied between the websites. Of note, contact information was always provided at the same place in the homepage (phone numbers have been blurred here for privacy reasons, photo-editing made from screenshots taken in June 2021).

The federation uses a website framework that brings together online resources that is reproduced in most cooperatives’ websites (Figure 1). While a common layout and logo are adopted by most of the cooperatives, there are few distinctions between the websites, allowing a certain degree of personalizing in the online presentation of the various individual cooperatives. Although widely used, the official logo of the federation (a deconstructed circle of triangles with one part standing out, Figure 1) is not present in all the cooperatives. Indeed, 8 out of 21 cooperatives do not feature the common logo in their online visual identity (Table 1). Regarding the layout of the websites, most cooperatives adopt the same general layout as the federation’s website. This design includes a reproduction of the federation’s logo in the bottom of the main page, as a way to further increase the identification of the cooperative with the federation. This general framework design also includes links to the cooperative’s social media pages at the bottom of the main page. However, while the design is conserved across individual websites, details allow the customization of each member of the federation. For instance, while the federation website has gray-green headings, some cooperatives use a different color code – with red, black, green, yellow, or blue headings (Figure 1). Therefore, even though all members of the federation agree upon a similar format, they do exert individuality in their choices of color. Having the same logo and overall design come with some advantages for the cooperatives, both from an internal perspective (as doing so obviously reduce costs related to web design and general marketing products), and from an external perspective (with customers identifying more quickly and easily a distinct trademark). Therefore, the choice of using a shared logo and design, but with options for customization in the color code, offers a good equilibrium between identity (of being a specific, locally grounded cooperative) and identification (as being part of a cooperatives’ federation). Interestingly, few exceptions to this rule exist. Indeed, in addition to the eight members not using the logo, three members of the federation elected to have a distinctive layout for their website (Table 1). In the case of these institutions, the membership to the federation is less obvious to the website’s visitors.

**Table 1.** List of the federation cooperatives’ websites.

Cooperative’s Name	Website	Logo	Layout
Alliance funéraire du royaume	https://www.afdr.coop/	No	Yes
Centre Funéraire coopératif du granit	https://www.cfgranit.qc.ca/	Yes	No
Coopérative funéraire Brunet	https://coopfbrunet.com/	Yes	Yes
Coopérative funéraire de l’Estrie	https://www.coopfuneraireestrie.com/	Yes	Yes
Coopérative funéraire de l’Outaouais	https://www.cfo.coop/	No	Yes
Coopérative funéraire de la région de coaticook	https://www.salonfunerairecoaticook.com/	Yes	Yes
Coopérative funéraire de la rive-Nord	https://cooprivenord.com/	Yes	No
Coopérative funéraire de Saint-Jean-de-Matha	https://www.coopfunerairestjeandematha.com/	No	Yes
Coopérative funéraire des Deux rives	https://www.coopfuneraire2rives.com/	Yes	Yes
Coopérative funéraire des Eaux Vives	https://www.eauxvives.ca/	Yes	Yes
Coopérative funéraire des Laurentides	https://www.coopfunerairelaurentides.org/	Yes	Yes
Coopérative funéraire du Bas-Saint-Laurent	https://www.cfbsl.com/	No	No
Coopérative funéraire du Fjord	https://fjord.coop/	No	Yes
Coopérative funéraire du grand Montréal	https://www.cfgrandmontreal.com/	Yes	Yes
Coopérative funéraire du Témiscamingue	https://www.cftemiscamingue.com/	Yes	Yes
Coopérative funéraire Haute-côte-Nord – Manicouagan	https://www.cfhcn.ca/	Yes	Yes
Maison funéraire de l’Amiante	https://www.mfamiante.coop/	Yes	Yes
Résidence funéraire de l’Abitibi-Témiscamingue	https://www.residence-funeraire.coop/	No	Yes
Résidence funéraire Lac-St-Jean	https://www.residencefunerairelacstjean.com/	No	Yes
Résidence funéraire Maska	https://www.rfmaska.coop/	Yes	Yes
Résidence funéraire de Lanaudière (Auxiliary member)	https://rflanaudiere.com/	No	No

**Figure 2.**
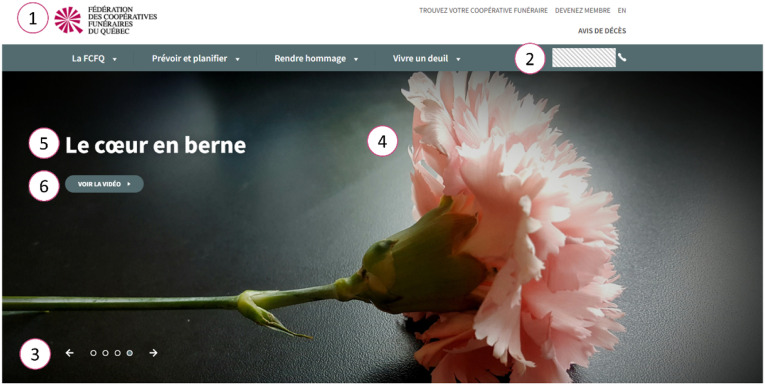
Homepage display screen. This figure presents a screenshot of one of the panels from the homepage moving display screen on the federation’s website. Interesting elements from the design are indicated with numbers: 1) the federation’s logo, 2) the federation’s contact information (phone number has been blurred here for privacy reasons), 3) display of slideshow controls (visitors can move from one panel to the others), 4) iconography choice varies from one panel to the other, 5) panel’s title or a comforting quote, 6) in some cases a hyperlink to multimedia resources (such as a video) can also be provided (screenshot taken in December 2021).

The choice of iconography on the websites’ main page also varies from one website to another. The federation’s website main page presents, below the headliners, a four-panel moving display screen (Figure 2). Each of these display screens presents a general illustration pertaining some emotional weight (hands handling each other, flowers, comforting symbols), a comforting quote (e.g., “*Présent à chaque instant*” i.e., present at all times) and optionally a link to multimedia material (typically a video or a resourceful website). While typically retaining the same structure as the main page of their own websites, the individual cooperative might or not replace one or more of the supporting pictures of the welcome display screens by iconography specific to their own cooperative identity (e.g., a picture of their building, their installations, or of the specific features of their geographical area). For instance, the “*Coopérative funéraire du Fjord*” (located near the Saguenay River Fjord) present peculiar iconography consisting of pictures of the iconic landscapes of the geographical area it deserves. Some websites also present moving display screens with more than four panels. The characteristics of the websites of the federation and its members (shared display, easy-to-find contact and location information, explicit subheading categories), as well as the design choices (comforting images and words), echo what has been observed for other funeral industry websites (Coetzee et al., 2014), and is likely to enhance the quality of the consumers’ experience.

Among the associate cooperatives, only one website presented the same layout as the federation (Funeral Co-operative of Ottawa Inc.) and three websites presented their association with the federation in a distinct section (“*Coopérative funéraire de Nantes*” (France), Funeral Cooperative Passage (PEI, Canada), The Co-op Funeral Home of People’s Memorial (USA)). Of note, a few clicks are needed in order to access this information, which was either located in an affiliations section (e.g., Funeral Cooperative Passage, The Co-op Funeral Home of People’s Memorial), or in a history section (e.g., “*Coopérative funéraire de Nantes*”). Interestingly, the Funeral Co-operative of Ottawa Inc website presents multiples visual similarities with most of the cooperative’s websites that adopt the general design framework, making this associate member cooperative harder to visually distinguish from actual member cooperatives.

### Online presence: Content analysis

Not surprisingly, all of the members’ websites were in French. However, two of them also proposed an English version. Of note, these two cooperatives were located in geographical areas of the province where French and English-speaking populations were more evenly distributed (i.e., Montreal and Outaouais). Yet, when available, the English version was significantly less complete and informative than the French version. This vastly monolingual characteristic of the federation obviously limits the audience of the federation to French speakers. However, English speakers can access funeral services through other companies not related to the federation, some of them arguably tailored to the needs of the English-speaking populations. The languages used in the websites of the associate cooperatives were more varied, and included French (2 websites), English (4 websites), bilingual French-English (2 websites), and Spanish (2 websites).

The websites using the common framework were divided in four headings: “*Prévoir et planifier*” (i.e., planification), “*Rendre hommage*” (i.e., pay tribute), “*Vivre un deuil*” (i.e., experiencing bereavement), “*Votre coopérative*” (i.e., the cooperative’s presentation). These four headings contain links to multiple relevant pages related to the overall theme. Therefore, numerous clicks are needed to browse the totality of the websites’ content. While a lot of information can be found in the websites, skills are needed to locate specific information (Guitton, 2015). In the context of a service related to health, which is touching people from all socio-economical origins, and likely people more vulnerable in terms of digital literacy (e.g., older adults), this might reduce the immediate actionability of the site for the users (Beaunoyer et al., 2017). This brings some nuance to the aforementioned initial ease of access of the websites themselves through online search engines (Beaunoyer, Hiracheta Torres, et al., 2020), and of their homepages through the characteristics of their visual features. For instance, the funeral services are not necessarily put forward, with minimal information being disclosed. While the federation claims to offer lower prices for funerals both to members and non-members (or with the “*Programme solidarité*” which proposes reduced fees for funeral rituals for deceased children), information on the prices of the various services offered is hard to locate on the cooperatives’ websites, when not simply absent. One cooperative does provide a presentation of forfeit prices for various types of ceremonies in a page advertising prior arrangement. However, it is clear that customers need to ultimately contact the federation member to get an idea or an estimate of the prices.

Most of the cooperatives’ websites present a link to an online platform for funeral planification called “*Espace Famille*” (i.e., family space). This platform constitutes a private and personalized space to obtain support in funeral planification throughout all the steps of the process. This tool serves to ease the communication between family members and funeral team, and to gather information in the same space (Coopérative funéraire des Deux Rives, 2018). This also allows for family members who are geographically far from the funeral home to be consulted. In addition to this institutional support space, the websites typically provide a link to an online grief support service (“*La Gentiane*”), which will be discussed below. Death notices were present, yet were very similar to traditional printed death notices, with basic information about the deceased family members and information on the funeral setting (when and where) being provided, content similar to those found on specialized sites such as legacy.com. Of note, bereaved or closed ones could publicly share their condolences through a commentary feature presented alongside the death notice. In the present case, death notices seemed to be often generated and used to inform the social network of the deceased individuals through social media. Interestingly, and despite the historically heavy influence of the Catholic Church and religious rituals in the Quebec society, little or no religious symbols or references were identified in the websites. Finally, cooperatives’ websites also present an online shopping space, where visitors can buy commemorative merchandise specifically for a deceased received at the cooperative.

## Building a community

### Death education and grieving support

One of the principles clearly emphasized by the funeral cooperative movement – and by the federation itself – is that one of the community-based missions of the funeral industry is to promote death literacy and death education among the population (Fédération des coopératives funéraires du Québec, n.d.-b). Whilst in a traditional model, this promotion of death education was de facto done locally, Internet allows for that to be done more broadly – within the inherent limitation of access to technology, and of language barriers. Another difference that is enabled by online presence of the funeral industry – and that is actualized in the federation suite of online resources – is the fact that, in the pre-Internet era, funeral industry-powered death education was typically occurring during a business/customer relationship, while in the digital era, the opportunities for the population to be exposed to death education material coming from the funeral industry are considerably more numerous – as evidenced by the ease with which the federation’s websites can be located online (Beaunoyer, Hiracheta Torres, et al., 2020).

Information on death education and death education services is presented through multimedia on the federation website and on the websites of the individual cooperatives, both in written format and in multimedia format (including videos). Interestingly, one cooperative sponsored a web series hosted by two local comedians in which humor, testimonials from local famous personalities, and interventions from the cooperatives’ employees were used to demystify death and funeral rituals in a speech designed for death education purposes (Coopérative funéraire du Grand Montréal, n.d.). Beside the information provided in the various pages of the websites of the federation and of its individual – regular or associate – members, the federation offers various and specific online or hybrid resources to further death education. For instance, the federation publishes a magazine entitled “*Profil*”, freely available online, which is used to communicate with the population about topics related to funerals, grief, and bereavement. Each issue includes an interview with a known personality of Quebec who speaks openly about a bereavement they experienced. This death education approaches is interesting as it might mobilize the parasociality relations between individuals and celebrities, in order to raise awareness toward grief and bereavement experience (Beaunoyer & Guitton, 2021, Park & Hoffner, 2020). Furthermore, a paper copy of this magazine can be provided in some cases in the different cooperatives.

In addition, most of the funeral cooperatives of the federation organize informational meetings, conferences nights, reflexional groups, and workshops (Fédération des coopératives funéraires du Québec, n.d.-b). These activities serve the objective of demystifying death through various informative themes related to personal (getting older, death and children, grief) and legal issues (end-of-life wishes, will, succession). The federation’s websites advertise these activities, thus proposing them to a potentially larger audience than the people who are already customers of the cooperatives.

An interesting death education resource brought forth by the federation’s online suite is the autonomous website named “*La Gentiane*” (https://www.lagentiane.org/). “*La Gentiane*” is a website which was first designed in the 1990s by a couple from Quebec. The first installment of this website was a forum developed in order to share support about their own grief experience. The forum became quite popular, and soon became an important source of potential peer support related to grief and mourning. The website got purchased by the federation in 2006, but the founding authors remained involved in the development of content and in the website maintenance (Tessier, 2007). Beside its historical feature – a discussion forum with over 4000 members and over 200 000 comments, the website contained three main sections. The first section provides 11 general articles covering themes such as helping a bereaved person, seeking help, losing of a child, child bereavement, or the stages of grief. The second section allows to share creative material, such as poems, letters, or testimonies. Finally, the third section gathers various articles classified within seven categories (grief and bereavement, funeral rituals, death, practical questions, testimony, other). Interestingly, and although the forum of “La Gentiane” was the only forum owned by a funeral institution that was easily retrieved from an exhaustive mapping of grief and death online resources (Beaunoyer, Hiracheta Torres, et al., 2020), the forum itself closed in the last week of August 2019 – and later became inaccessible. While it was heavily frequented 10 years ago, the postings increasingly decreased across the years – reaching even 0 in the last few months before the forum was closed. The change in the dynamics was acknowledged by the administrators of the forum that consequently decided to change the medium used for the peer support. A Facebook group was created instead, which might suggest that social media platforms might represent a better vector for peer support about grief, as demonstrated for other health issues (Lazard et al., 2021; Zhang et al., 2021).

The death education initiatives conducted by the federation contribute more generally to prepare the families facing the funeral planning. Although it is impossible from the present descriptive analyses to infer the degree of penetration of these death education programs in the population, it is reasonable to think that the reach extends *a minima* to a proportion of the population corresponding to the federation’s market shares. In addition, people could access some of these resources (e.g., informational web pages) without being a customer, as some of these services were free. Finally, when exploring the forum, some contributions were clearly coming from individuals located outside of the province – notably people from European French-speaking countries. This points to an international audience for the grief online support resources hosted by the federation. Incidentally, this reinforces the view that cyberspace supports communities that transcend physical geographical barriers. Taken together, this implication of the federation in death education extends the function of the federation as an actor within the community.

### Witnessing the evolution of rituals

Funeral industries offer numerous services, ranging from the body curation to the funeral planning. However, these services are not necessarily those that are put forward on the cooperatives’ websites. Although some cooperatives’ websites formally present the various funeral services offered, they typically suggest potential customers to directly contact the cooperative to receive more information. Instead, the websites focus on other services – specifically services combining memorialization and grief support purposes. In this view, the websites seem to acknowledge a commitment toward other societal values, illustrating how the federation presence can go beyond the single delivery of funeral services and extend throughout the grieving process.

Among the initiatives bearing a societal value advertised throughout the federation websites, we can mention an ecological memorialization program called “*Programme Héritage*” (i.e., inheritance program). This program is presented on the federation main website and on most of the members’ websites. It involves planting trees in memorial to each deceased received at the cooperative (Fédération des coopératives funéraires du Québec, n.d.-c). Despite being a way to memorialize a loved one, the deceased memory is honored through an action that not only contributes to protecting life – symbolizing a form of life beyond death –, but is also part of a global, and societal, action to protect the environment and to reduce the ecological footprint of human activities related to funerals (e.g., vehicles used for deceased transportation). In 2020, 95 560 trees were planted to commemorate the deceased, and 64 896 more to cover 100% of their ecological footprint (Fédération des coopératives funéraires du Québec, n.d.-c). In this case, memorialization is combined with ecological values both on an individual level (individual meaning of the tree as an extension of the deceased life) and on a global one (social responsibility of reducing its ecological footprint). This not only witnesses the evolution of existing mourning rituals, but also contributes to the emergence of new forms of rituals, which, while taking place in physical spaces, are powered by virtual spaces.

The rituals conducted by the cooperatives can be further personalized with and within the “family space” (online platform we presented earlier in this text). Indeed, this platform is not only used to help families during funeral planning, but also to provide them a space for actively participating in the creation of unique memorialization content related to the deceased (Alliance funéraire du Royaume, n.d.-a). This online tool is part of a broader approach aiming to add value for both the funeral industry and the families by extending mourning rituals to a professionalized cyberspace. This approach to customers is also evidenced by a formation program given to counselors and funeral directors centered on the importance of meaningful rituals (Fédération des coopératives funéraires du Québec, n.d.-d). For the federation, giving meaning to the funerals implies more than to be up to date with digital or non-digital funeral services. It also involves promoting the implication of the bereaved in the funeral planification and organization, as well as in the memorialization process. However, considering that not everyone is familiar and competent with Internet use (Beaunoyer, Dupéré, & Guitton, 2020), the use of this tool still remains optional. Indeed, important differences exist between individuals regarding their capabilities to access technological devices, and their degree of competence needed to benefit from using technology, a phenomenon referred to as digital inequalities (Beaunoyer, Dupéré, & Guitton, 2020; DiMaggio & Hargittai, 2001). More than a simple biphasic “digital divide”, digital inequalities can be represented through a multi-dimensional continuum of factors including – but not limited to – socio-economic status, age, education level, geographical location (Beaunoyer, Dupéré, & Guitton, 2020; Robinson et al., 2019). Interestingly, this is a striking example of how digital inequalities can interfere beyond death, with families having more digital skills potentially able to reach higher levels of individualizing their rituals, and even to include other relatives who would not be able to be physically present (Beaunoyer & Guitton, 2021). In the context of remembrance, this shows how social structures can encourage the creation of new rituals. In this sense, technology both enables and mediates the emergence of new ways to memorialize deceased people, ultimately embracing a hybrid reality, encompassing both offline and online spaces as commemoration spaces, while still being supervised by the industry. The active implication of family members in the elaboration of a funeral service uniquely conceived for the deceased, improved by the “family space”, thus promotes empowerment and action in the grieving process.

### Creating a sense of community

The online presence of the federation’s members suggests other ways through which a sense of a community might arise, beside the outcome of the funeral services from any funeral institution that could create a sense of community. Almost all of the federation’s individual cooperatives have at least one social media account, notably on Facebook. Indeed, the federation and all membered cooperatives but one (“*Coopérative funéraire de Saint-Jean-de-Matha*”) have a Facebook account. However, the social media presence of the individual cooperatives is unequal in numerous ways. First, the number of posts shared by the federation or by its membered cooperatives greatly varied. For instance, while the mean number of posts shared in the last 30 days (November 23 to December 23, 2021), was 13,67 posts (SD: 19.05), the minimal number of posts shared by a cooperative Facebook page was of 0 (4 cooperatives) and the maximum number of posts shared was of 92 (“*Coopérative funéraire du Bas-Saint-Laurent”).* As a comparison point, the federation itself shared 12 posts. The material posted by the federation was also mostly reposted or shared on the Facebook feed of the individual cooperatives. Furthermore, the cooperatives that shared the most content on their Facebook page, were also those publishing the most death notices of the deceased they were received. Second, the number of followers of the federation and of the cooperatives also varied. While the mean number of followers is 635.5 (SD: 577.77), the minimal number of followers of a Facebook account was of 16 (“*Résidence funéraire de l’Abitibi-Témiscamingue*”) and the maximal number of followers was of 2215 (“*Coopérative funéraire du Grand Montréal*”). Third, the federation and some cooperatives have other types of social media accounts, such as Twitter (the federation and four cooperatives), Instagram (“*Coopérative funéraire des Deux Rives*”), LinkedIn (the federation and “*Coopérative funéraire du Grand Montréal*”), and YouTube (“*Résidence funéraire Lac-St-Jean*”). The federation is also present on Pinterest and Vimeo. However, those accounts are typically poorly fed, or not regularly updated. Therefore, the cooperatives’ sense of community does not appear to primarily rely on social media presence, but might be more related to other forms of actions.

Funeral industry engagement in community activities and sponsorship of programs contribute to developing the establishment participation in the local goodwill activities, which could lead to word-of-mouth advertising and increase trust in the funeral home (Beard & Burger, 2020). Through their websites, each cooperative is given space to express their individuality in relation to their role in the community – or at least to the role they perceive or want to display. While all cooperatives mostly offer the same common services, some also offer local services tailored to their community that are not necessarily offered elsewhere. Indeed, browsing the various cooperatives’ websites reveal information regarding how each position their social implication within the community. This implication can take the form of discounts on funeral expenses for the members, donations, and sponsorships. For instance, the website of one cooperative presents a social and economic implication in the community that translates into donations to various organizations in their territory, counting in thousands of dollars each year (Alliance funéraire du Royaume, n.d.-b). The news section of the federation website (which includes all member cooperatives news) also features community donation engagements or realizations (Coopérative funéraire des Deux Rives, 2020; Coopérative funéraire de Saint-Jean-de-Matha, 2021). Another form of social implication that was promoted through some cooperatives websites is the possibility for individuals or organizations to rent space for a minimal price (Coopérative funéraire des Deux Rives, n.d.).

By their implication in death education interventions, and in various programs such as their ecological memorialization program or their philanthropic activities, the cooperatives of the federation promote and advertise their social implication in their communities through their online presence. By doing so, the federation’s membered cooperatives contribute to bringing back a sense of community – and therefore a sense of life – to something that was traditionally perceived as morbid by going beyond the single funeral rituals. Indeed, the cooperatives present themselves as an important part of the community and intervene not only in end-of-life contexts, but also in life ones.

## Handling rituals in a sanitary crisis context

The COVID-19 pandemic, emerging in March 2020, challenged all parts of the social systems around the world. As a means to contain the propagation of the virus, the Quebec government – as most governments worldwide – imposed several social distancing measures that impacted the funeral industry. These measures varied according to the cyclic outbreaks that happened in the province of Quebec, as it did in other regions of the world. While these restrictions increased the dependency upon digital technologies, the capability – and thus the actual possibility – to participate and to retain benefits from digital funeral rituals is closely related to digital inequalities (Beaunoyer, Dupéré, & Guitton, 2020). During the first outbreak, the federation chose to cancel and postpone funeral ceremonies and activities (Fédération des coopératives funéraires du Québec, 2020), while other independent industries limited gatherings to two persons at the same time or moved online most funeral events and contacts (Nadeau, 2020). The activities were gradually reinstated during the following waves with the application of strict measures such as hand disinfection, physical distancing, wearing a mask, prohibition of physical contacts such as hugging and hand shaking, and reduction of the maximal capacity of people attending the ceremonies.

**Figure 3.**
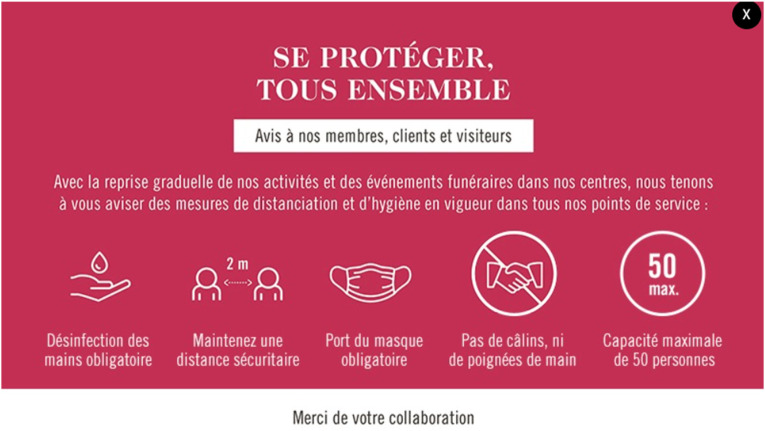
Pop-up window presenting the COVID-19 sanitary measures. This figure is an example of a pop-up window appearing when visiting the website of one of the cooperatives, presenting the COVID-19 sanitary measures in place at the cooperative (in this case, the “Coopérative funéraire du Grand Montréal”). The pop-up window presents the principals sanitary measures to follow when visiting their establishments (texts in French, screenshot taken in December 2021).

While dealing with the pandemic, clear communication between the cooperatives and their customers was and is essential. The federation used multiple communicative features in their websites to inform visitors and customers of the services updates. When one of the cooperatives’ websites is accessed, a pop-up window appears indicating the basic hygiene measures that visitors of service points need to follow (Figure 3). The measures presented in this pop-up window varied through time alongside the governmental recommendations. The news section of the federation website proposed multiple articles informing the reinstatement of various funeral services in the first half of the year 2020. These articles originated both from the federation itself or from the cooperatives. For instance, the “*Coopérative funéraire du Grand Montréal*” published a piece of news on May 29, 2020, informing that columbarium visits were possible again by appointment for one or two persons at the same time (Coopérative funéraire du Grand Montréal, 2020).

Modifying the welcoming conditions of the guests attending in situ funerals was, however, not sufficient to answer the crisis. Indeed, giving the pace of infections, a lot of funeral ceremonies had to go virtual. Therefore, the federation also updated their offer of digital services to answer the needs of bereaved family while respecting governmental and sanitary restrictions in conducting funeral services. Consulting the news section of the federation website allowed to find more information regarding the adaptations made to the digital rituals in the pandemic context. The obituary pages were updated to support more interactions. Notably, the obituary pages proposed several new virtual services to complement the redaction of sympathy messages. Among them were both services free of charge, including the possibility to add a photo tribute (Coopérative funéraire des Deux Rives, 2020; Coopérative funéraire de l’Estrie, 2020) or to light a virtual candle, and services offered for a limited price, such as a tree plantation in memorial of the deceased (Coopérative funéraire des Deux Rives, 2021). Online diffusion of funeral ceremonies became more accessible in numerous cooperatives (Coopérative funéraire des Eaux Vives, 2020; Résidence funéraire Maska, 2021). Updates in digital services were not only made with regards to typical funeral services and memorialization, but also for grief support services and death education initiatives. Indeed, some cooperative offered virtual meetings for the bereaved (Coopérative funéraire de l’Outaouais, 2020). The death education mission was also pursued and adapted to the new digital reality by replacing the traditional annual public conferences with virtual conferences (Coopérative funéraire des Deux Rives, 2020).

In accordance with the federation supportive online presence, the federation general framework (used for most of the cooperatives’ websites) was updated in response to the pandemic. Thus, the subsection “*Le deuil en période de confinement*” (i.e., mourning during confinement) was added to the section “*vivre un deuil*” (“living a loss”). The content presented in this section was further divided in three subsections: “*Le deuil et les rituels*” (i.e., grief and rituals), “*S’entraider*”, (i.e., helping each other), and the “family space”. In the section “grief and rituals”, the person can first access a downloadable guide for grief support in periods of isolation. This guide contains advice on the type of rituals that can be conducted at home (e.g., dedicating a small space to the deceased loved one with pictures and few objects) and alternatives to receive support in the absence of traditional funeral rituals (e.g., share memories and express emotions to friends and family by phone and through social media). The last page of the guide presents some online resources offered by the federation such as “*La Gentiane*” and instructive readings that can be found on the cooperative websites. Furthermore, two short videos are displayed where supporting grief quotes scrolls along with suiting music and one video presenting advice from a counselor explaining how to write a tribute. Some quotes, used in the Facebook pages of the cooperatives, are also presented. An audio interview with one of the founders and current managers of “*La Gentiane*” from March 30, 2020, discussing about grief and funeral rituals in a pandemic context is also accessible directly on this same page. The section “helping each other” includes links for the federation support resources of “*La Gentiane*”, the website and the Facebook community, as well as for phone support. While “*La Gentiane*” offers online support, phone support is not directly available online and the bereaved who seeks it needs to contact its local cooperative to receive this type of support free of charge. Finally, the link for the section “family space” leads to a description of the service that is contextualized to the pandemic situation.

While the federation initial communication around COVID-19 suggested that the funerals were only held up until the end of the crisis, time made abundantly evident that the crisis would not pass quickly enough for the bereaved to simply wait to accomplish funeral ceremonies. Therefore, multiple adaptations were made to the services, as presented in the paragraphs above. However, given that after two years the situation is still unstable with the waves of outbreak succeeding each other, it is likely that at least some of the restrictions to funeral services will remain in a foreseeable future. In this view, the evolution of the content of the federation and its cooperatives’ websites testifies to the forced adaptation that the funeral industry had to undergo.

## Conclusion

The analysis of the online presence of Quebec federation of funeral cooperatives is highly informative. Of note, the observations presented here are purely descriptive. Thus, this case study should not necessarily be generalized to funeral companies located in Quebec that would not belong to the federation. While this case might appear as specific, some elements suggest that this example might not be that singular and could potentially inform other phenomena related to the online presence of funeral industry. Quebec geography and demography are characterized by a large territory housing a rather small population, which suggests that online services could be increasingly important in the near future. The pandemic gave a clear taste of what it could be like if the physical boundaries could not be crossed anymore. Yet, the influence of the federation over the funeral industry might not be strictly limited to the large geographical boundaries of Quebec. Indeed, associated cooperatives also extend the movement to the rest of Canada, to other countries, as well as to other continents.

The findings illustrate how the funeral industry is evolving with the digitalizing of the society, alongside the needs of the various local communities they are meant to serve. The online presence of the federation and its cooperative contributes to put forward the principles of education, training, memorialization, and grief support as well as a concern for the community. As such, the websites of the federation and its cooperatives are witnessing the promotion of a dialog encouraging grief and death literate society. Future studies should document further how the Quebec population participates to and benefits from this integrated hybrid dialog. Once the crisis is over, the online services emerging from the response to the crisis will likely remain, as tools supporting the displacement of funeral rituals from physical spaces to hybrid modalities. This crisis will have long-lasting impacts on the funeral industry as it accelerated this movement toward hybrid spaces for mourning. Future research will need to document the impacts of those changes on the communities that experience them firsthand, especially among digitally disadvantaged populations. As the near future will likely be welcoming more technological innovations, i.e., augmented reality, the access to funeral services will differ among the population, leaving vulnerable populations even more at bay.

## References

[bibr1-00302228221111936] Alliance funéraire du Royaume (n.d. a). Espace-famille – planification décès. Retrieved June 8, 2021, from https://www.afdr.coop/services/espace-famille-planification-deces/

[bibr2-00302228221111936] Alliance funéraire du Royaume (n.d. b). Implication sociale. Retrieved June 2, 2021, from https://www.afdr.coop/votre-cooperative/implication-sociale/

[bibr3-00302228221111936] Arnold M. Gibbs M. Kohn T. Meese J. Nansen B. (2018). Death and digital media. Routledge. 10.1080/07481187.2018.1522387 30623744

[bibr4-00302228221111936] Beard V. R. Burger W. C. (2017). Change and innovation in the funeral industry. Omega – Journal of Death and Dying, 75(1), 47–68. 10.1177/0030222815612605 28395641

[bibr5-00302228221111936] Beard V. R. Burger W. C. (2020). Selling in a dying business: An analysis of trends during a period of major market transition in the funeral industry. OMEGA – Journal of Death and Dying, 80(4), 544–567. 10.1177/0030222817745430 29235385

[bibr6-00302228221111936] Beaunoyer E. Arsenault M. Lomanowska A. M. Guitton M. J. (2017). Understanding online health information: Evaluation, tools, and strategies. Patient Education and Counseling, 100(2), 183–189. 10.1016/j.pec.2016.08.028 27595436

[bibr7-00302228221111936] Beaunoyer E. Dupéré S. Guitton M. J. (2020). COVID-19 and digital inequalities: Reciprocal impacts and mitigation strategies. Computers in Human Behavior, 111, 106424. 10.1016/j.chb.2020.106424 32398890 PMC7213963

[bibr8-00302228221111936] Beaunoyer E. Guitton M. J. (2021). Cyberthanathology: Death and beyond in the digital age. Computers in Human Behavior, 122, 106849. 10.1016/j.chb.2021.106849

[bibr9-00302228221111936] Beaunoyer E. Hiracheta Torres L. Maessen L. Guitton M. J. (2020a). Grieving in the digital era: Mapping online support for grief and bereavement. Patient Education and Counseling, 103(12), 2515–2524. 10.1016/j.pec.2020.06.013 32591255

[bibr10-00302228221111936] Coetzee C. A. Maree T. (2014). The marketing of an unsought service through an unobtrusive medium: A content analysis of the websites of members of the national funeral directors association of South Africa. Communicare: Journal for Communication Sciences in Southern Africa, 33(1), 35–55.

[bibr11-00302228221111936] Coopérative funéraire de l’Estrie . (2020, June 14). L’avis de décès : Le point *central de la communication avec les proches*. https://www.coopfuneraireestrie.com/nouvelles/avis-deces-point-central-communication-avec-3294/

[bibr12-00302228221111936] Coopérative funéraire de l’Outaouais . (2020, April 24). Rencontres virtuelles thémat*iques avec Entraide-Deuil Outaouais*. https://www.cfo.coop/nouvelles/rencontres-virtuelles-thematiques-avec-entraide-deuil-3216/

[bibr13-00302228221111936] Coopérative funéraire de Saint-Jean-de-Matha . (2021, March 19). Œuvre caritative. https://www.coopfunerairestjeandematha.com/nouvelles/ouvre-caritative-3475/

[bibr14-00302228221111936] Coopérative funéraire des Deux Rives . (2018, Febuary 7). Nouveauté! – espace Famill*e*. https://www.coopfuneraire2rives.com/nouvelles/nouveaute-espace-famille-2688/

[bibr15-00302228221111936] Coopérative funéraire des Deux Rives . (2020, December 9). La Coopérative remet 10 000$ en *dons spontanés à 10 organismes sur son territoire*. https://www.coopfuneraire2rives.com/nouvelles/cooperative-remet-10-000-dons-spontanes-3389/

[bibr16-00302228221111936] Coopérative funéraire des Deux Rives . (2020, September 18). Nouveauté : Conférences en ligne*!* https://www.coopfuneraire2rives.com/nouvelles/nouveaute-conferences-ligne-3354/

[bibr17-00302228221111936] Coopérative funéraire des Deux Rives . (2020, May 4). La Coopérative au service *des familles même si les funérailles sont suspendues*. https://www.coopfuneraire2rives.com/nouvelles/cooperative-service-des-familles-meme-les-3226/

[bibr18-00302228221111936] Coopérative funéraire des Deux Rives . (2021, May 5). Nouveauté – lampions virtu*els et arbres commémoratifs*. https://www.coopfuneraire2rives.com/nouvelles/nouveaute-lampions-virtuels-arbres-commemoratifs-3520/

[bibr19-00302228221111936] Coopérative funéraire des Deux Rives (n.d.). Location de nos locaux. Retrieved June 7, 2021, from https://www.coopfuneraire2rives.com/votre-cooperative/location-nos-locaux/

[bibr20-00302228221111936] Coopérative funéraire des Eaux Vives . (2020, November 17). Un nouveau service de diffusion *web à la Coopérative des Eaux Vives*. https://www.eauxvives.ca/nouvelles/nouveau-service-diffusion-web-cooperative-funeraire-3380/

[bibr21-00302228221111936] Coopérative funéraire du Grand Montréal . (2020, May 29). Ré-ouverture des columbari*ums*. https://www.cfgrandmontreal.com/nouvelles/ouverture-des-columbariums-3265/

[bibr22-00302228221111936] Coopérative funéraire du Grand Montréal (n.d.). Épisodes La mort nous va si bien. Retrieved December 17, 2021, from https://www.cfgrandmontreal.com/votre-cooperative/la-mort-nous-va-si-bien/

[bibr23-00302228221111936] Cyrus J. W. (2014). A review of recent research on internet access, use, and online health information seeking. Journal of H, 14(2), 149–157. 10.1080/15323269.2014.888630

[bibr24-00302228221111936] DiMaggio P. Hargittai E. (2001). From the “digital divide” to “digital inequality”: Studying internet use as penetration increases. Center for Arts and Cultural Policy Studies, 15, 1–23. Princeton University 10.1002/bem.20484

[bibr25-00302228221111936] Fédération des coopératives funéraires du Québec . (2013, January, 31). Une industrie qui s'adapte au temps. https://www.fcfq.coop/nouvelles/une-industrie-qui-adapte-temps-1963/

[bibr26-00302228221111936] Fédération des coopératives funéraires du Québec (2020, March 16). Les coopératives funéraires *reportent les funérailles*. https://www.fcfq.coop/nouvelles/les-cooperatives-funeraires-reportent-les-funerailles-3146/

[bibr27-00302228221111936] Fédération des coopératives funéraires du Québec (n.d. a). Le mouvement des coopératives funéraires au Québec en quelques chiffres. Retrieved December 14, 2021, from https://www.fcfq.coop/la-federation/

[bibr28-00302228221111936] Fédération des coopératives funéraires du Québec (n.d. b). Éducation. Retrieved December 22, 2021, from https://www.fcfq.coop/services/education/

[bibr29-00302228221111936] Fédération des coopératives funéraires du Québec (n.d. c). Le programme Héritage. Retrieved December 14, 2021, from https://www.fcfq.coop/services/projet-heritage/

[bibr30-00302228221111936] Fédération des coopératives funéraires du Québec (n.d. d). Funérailles riches de sens. Retrieved June 7, 2021, from https://www.fcfq.coop/services/symphonie/

[bibr31-00302228221111936] Guitton M. J. (2011). Immersive role of non-required social actions in virtual settings: The example of trade role-play in the Second Life Gorean community. Design Principles and Practices, 5(1), 209–219. 10.18848/1833-1874/cgp/v05i01/38012

[bibr32-00302228221111936] Guitton M. J. (2012). Living in the hutt space: Immersive process in the star wars role-play community of second life. Computers in Human Behavior, 28(5), 1681–1691. 10.1016/j.chb.2012.04.006

[bibr33-00302228221111936] Guitton M. J. (2015). Online maritime health information: An overview of the situation. International Journal of Maritime Health, 66(3), 139–144. 10.5603/IMH.2015.0028 26394313

[bibr34-00302228221111936] Hine C. (2000). Virtual ethnography. Sage.

[bibr35-00302228221111936] International Cooperative Alliance (n.d.). Cooperative identity, values & principles. Retrieved June 10, 2021, from https://www.ica.coop/en/cooperatives/cooperative-identity

[bibr36-00302228221111936] Lazard A. J. Reffner Collins M. K. Hedrick A. Varma T. Love B. Valle C. G. Brooks E. Benedict C. (2021). Using social media for peer-to-peer cancer support: Interviews with young adults with cancer. JMIR Cancer, 7(3), 1–13. 10.2196/28234 PMC8446843 34473063

[bibr37-00302228221111936] Mann C. Stewart S. (2000). Internet communication and qualitative research: A handbook for researching online. Sage.

[bibr38-00302228221111936] Marcus G. (1995). Ethnography in/of the world system: The emergence of multisided ethnography. Annual Review of Anthropology, 24(1), 95–117. 10.1146/annurev.an.24.100195.000523

[bibr39-00302228221111936] Metcalf P. Huntington R. (1991). Celebrations of death: The anthropology of mortuary ritual. Cambridge University Press.

[bibr40-00302228221111936] Ministère de la santé et des services sociaux (2022). Liste des entreprises de services funéraires – permis 2022. https://msss.gouv.qc.ca/professionnels/documents/domaine-funeraire/repertoire-directeurs-funerailles.pdf

[bibr41-00302228221111936] Nadeau J-F. (2020, April 4th). Pleurer ses morts, chacun chez soi. Le Devoir. https://www.ledevoir.com/societe/576439/funerailles-pleurer-ses-morts-chacun-chez-soi

[bibr42-00302228221111936] Nansen B. Kohn T. Arnold M. van Ryn L. Gibbs M. (2017). Social media in the funeral industry: On the digitization of grief. Journal of Broadcasting and Electronic Media, 61(1), 73–89. 10.1080/08838151.2016.1273925

[bibr43-00302228221111936] Northcott H. C. Wilson D. M. (2017). Dying and death in Canada (3rd ed.): University of Toronto Press.

[bib9003022282212] Park S. Hoffner C. A. (2020). Tweeting about mental health to honor Carrie Fisher: How #InHonorOfCarrie reinforced the social influence of celebrity advocacy. Computers in Human Behavior, 110, 106353. DOI:10.1016/j.chb.2020.106353

[bibr44-00302228221111936] Résidence funéraire Maska . (2021, June 8). Service de webdiffusion des cérémonies funéraires. https://www.rfmaska.coop/nouvelles/service-webdiffusion-des-ceremonies-funeraires-3313/

[bibr45-00302228221111936] Robinson C. Pond D. R. (2019). Do online support groups for grief benefit the bereaved? Systematic review of the quantitative and qualitative literature. Computers in Human Behavior, 100, 48–59. 10.1016/j.chb.2019.06.011

[bibr46-00302228221111936] Romanoff B. D. Terenzio M. (1998). Rituals and the grieving process. Death Studies, 22(8), 697–711. 10.1080/074811898201227 10346698

[bibr47-00302228221111936] Silver L. Huang C. Taylor K. (2019). Emerging economies, smartphone and social media users have broader social networks. Pew Research Center. https://www.pewresearch.org/internet/2019/08/22/social-activities-information-seeking-on-subjects-like-health-and-education-top-the-list-of-mobile-activities/

[bibr48-00302228221111936] Tessier A. (2007). Album souvenir 20e anniversaire : Historique de la Fédération des coopératives funéraires du Québec. Fédération des coopératives funéraires du Québec. https://www.fcfq.coop/media/FCFQ-historique.pdf

[bibr49-00302228221111936] Turner E. Rainie L. (2020, March 5) Most Americans rely on their own research to make big decisions, and that often means online searches. Pew Research Center. https://www.pewresearch.org/fact-tank/2020/03/05/most-americans-rely-on-their-own-research-to-make-big-decisions-and-that-often-means-online-searches/

[bibr50-00302228221111936] Zhang R. Bazarova N. Reddy M. (2021). May). Distress disclosure across social media platforms during the COVID-19 pandemic: Untangling the effects of platforms, affordances, and audiences. Proceedings of the 2021 CHI Conference on Human Factors in Computing Systems, Japan, 644, 1–15. 10.1145/3411764.3445134

